# Methodology and model-based DSS to managing the reallocation of inventory to orders in LHP situations. Application to the ceramics sector

**DOI:** 10.1371/journal.pone.0219433

**Published:** 2019-07-11

**Authors:** Raul Oltra-Badenes, Hermenegildo Gil-Gomez, Jose M. Merigo, Daniel Palacios-Marques

**Affiliations:** 1 Department of Business Organisation, Universitat Politècnica de València, Valencia, Spain; 2 Department of Management Control and Information Systems, University of Chile, Santiago, Chile; Shandong University of Science and Technology, CHINA

## Abstract

Lack of homogeneity in the product (LHP) is a problem when customers require homogeneous units of a single product. In such cases, the optimal allocation of inventory to orders becomes much more complex. Furthermore, in an MTS environment, an optimal initial allocation may become less than ideal over time, due to different circumstances. This problem occurs in the ceramics sector, where the final product varies in tone and calibre. This paper proposes a methodology for the reallocation of inventory to orders in LHP situation (MERIO-LHP) and a model-based decision-support system (DSS) to support the methodology, which enables an optimal reallocation of inventory to order lines to be carried out in real businesses environments in which LHP is inherent. The proposed methodology and model-based DSS were validated by applying it to a real case at a ceramics company. The analysis of the results indicates that considerable improvements can be obtained with regard to the quantity of orders fulfilled and sales turnover.

## Introduction

In a make-to-stock (MTS) strategy, production planning is based on a forecast of demand [1]. Subsequently, as real orders come in and are recorded, part of the available inventory is reserved for this order. Thus an amount of stock is allocated to the order, which then becomes unavailable for other possible uses, ensuring that the order can be fulfilled on the required date.

Optimal stock management and allocation of inventory to orders will ensure greater customer satisfaction, stock optimization, cost reductions and maximization of company turnover. However, at a particular time, this optimal allocation may become less than ideal, due to unforeseen circumstances within the company. In this case a reallocation of inventory to orders will be necessary.

Such a circumstance may occur in any industry, but it is particularly frequent in some areas of manufacturing, in which, due to the nature of their products and/or their production processes, there is a lack of homogeneity in the product (LHP).

LHP can be defined as “a lack of the homogeneity required by the customer for a particular product” and derives from the impossibility of manufacturing homogeneous products, either in a single batch or across batches produced by means of the same process. LHP represents a problem when a particular customer acquires several units from one or more batches of the product and requires them to be homogeneous because they are to be used, displayed, placed or consumed together [2].

LHP is characteristic in ceramics companies, whose manufacturing processes are such they produce products with a lack of homogeneity with regard to the colours (different tones) and sizes (different calibres). Thus, the stock of a particular product is fragmented, i.e. made up of different quantities of items displaying different “tone-calibre” combinations, which must be identified, stored and managed separately.

In this industry, when processing a particular order, the product delivered must be of the same tone and calibre, as the use of different tones and calibres can lead to undesirable results: these may be aesthetic, in the case of the use of tiles of the same colour but of different tones; or functional, as the use of tiles of different calibres on floors or walls may lead to uneven surfaces.

Therefore, the correct allocation of the different tone-calibre combinations of a particular product in the inventory to each order is of great importance. The LHP present makes optimal stock allocation a much more complex task and, as a result, a mathematical model for the reallocation of inventory to orders becomes necessary. However, the formulation of a mathematical model is not enough to solve the problem in a real environment. In order to execute the mathematical model properly in a company, it is essential to define a work methodology, establishing a series of steps to follow, and a system, that allows and facilitates implementation of that methodology efficiently.

This study examines and analyses the issue of the reallocation of stock in LHP settings by ceramics companies with orders with non-immediate delivery dates and which work to an MTS production strategy. In order to manage and solve this problem, a methodology and a computerized model-based DSS is presented here, which aims to facilitate the decision-making process, and allows integrating the mathematical model with the rest of the company's operating processes through its information system. Finally, the employment of the model-based DSS in a real case study, carried out in order to validate it, is described and the conclusions of the study are then presented.

## Literature review

Inventory allocation is a key aspect for the efficiency of the supply chain [3]. For this reason, it has been studied for decades. For example, already in the 1980s [4] studied a supply chain composed of a central warehouse and several regional warehouses, which supplied products to final customers. In that environment, they analyzed the best way to fully satisfy the demand, studying the options of maintaining a security inventory in the warehouse after the allocation of inventory to the intermediate retailers and allowing the transshipment between retailers to balance the subsequent inventories.

Nowadays inventory allocation continues to be an important research area. In recent works, this topic is studied from different perspectives and in very diverse sectors. For example, in military logistics there are interesting works proposing a network model for the improvement of inventory allocation and vehicle routes, including local repair and resupply capacity [5]. With a more social goal, the model developed in [6] minimizes the social costs of inventory allocation and studies the routes for sending critical supplies to populations in need after a catastrophe. In the production area, there´s a model for inventory allocation of bins to orders for LED-CM manufacturing plants [7]. This model is subsequently improved by introducing genetic algorithms [8].

More focused on the Supply Chain Management are other proposals about inventory allocation, as a reconfigurable supply chain network that optimizes the inventory allocation and transport route making the allocation in advance and thus being able to anticipate changes in transport routes [9]. The case of a manufacturer with multiple retailers, who reserves part of the inventory for a second replenishment so that it can replenish retailers' inventories optimally is presented in [10], while [11] develops a methodology of inventory allocation to limited shelf spaces in the case of a retailer in a supply chain with two suppliers of different characteristics (using a clothing store chain as example).

A model to optimize the production quantity of clinical drugs in the pharmaceutical industry, minimizing the costs of inventory allocation and production without lengthening the clinical trial is presented in [12]. With a similar objective [13] investigates the planning of inventory allocation in a supplier-manufacturer network, in order to determine the selection of the supplier, the amount of purchase, the level of inventory and the amount of allocation of each material in each period to minimize the total costs of the supplier-manufacturer network. Other works also focus on the selection of suppliers and the impact of the inventory allocation for this purpose [14;15].

Focusing on inventory allocation as "the set of decisions about the distribution of inventories from suppliers to customers when the supplier does not have sufficient stocks to serve demand in full for all customers" [3], numerous studies have attempted to optimise the allocation of inventory to orders, focusing the available-to-promise (ATP) concept.

There are also ATP allocation optimisation which maximise profits while taking penalties into account [16, 17], some others with different customer priorities and service strategies [18], and others which consider ATP allocation in stock-out situations [19]

A classification of ATP systems is established in terms of different concepts, such as the mode of functioning of the system (batch-mode or real time), the manufacturing strategy (MTO, MTS, ATO or pull or push) [20]. In [21] a determinist model is formulated for the ATP allocation of inventory to different “classes of customers”.

Notice that in [20] the reader is warned that ATP allocation to orders may be a “myopic” view of the situation, as the orders that arrive in a particular time period have resources allocated to them without the impact that this will have on the fulfilment of future orders being taken into account. Similar comments are made in [22], which provides an explanation of the problem of fulfilling orders in a system in which there is significant deviation between the forecasted and the actual demand, leading to stock-out situations when demand is higher and to excess of inventory situations when demand is weaker. The myopia of this view is also discussed in [23], in which an attempt is made to optimise the allocation of inventory to orders in order to minimise the number of shipments.

In the existing literature, there are also works that present DSS for the improvement of the ATP in relation to the inventory allocation. For example, [24] proposes a DSS that helps to establish due dates and prices to customers, while in [25] a DSS is presented that helps SMEs determine a feasible delivery date and verify its feasibility. [26] presents a DSS in real time to find an optimal allocation of available inventory. The DSS is incorporated into a steel bar production company's information system, while [27] proposes a DSS that integrates the acceptance of orders, allocation of delivery date and programming of orders, with the objective of maximizing the profit in a multiple order supply chain scenario.

In [28] a DSS is proposed with the aim of saturating capacity while increasing customer satisfaction, offering customers a final product that is very similar to the desired along in a shorter delivery time and therefore assigning the maximum possible ATP. The DSS generates product configurations that can be attractive to customers and suggests which versions of the products must be offered to customers in each period, in order to saturate the available capacity taking into account the attractiveness of the package of components for customers. A similar study can be found in [29] in which a DSS is proposed with the objectives of minimizing the delay of customer orders and optimizing the rate of use of the equipment, applied to the production lines in the semiconductor sector.

All of these studies represent an attempt to allocate inventory in the best possible way, but their perspective is also a “static” one. They are based on a given situation at a company and on a forecast about available inventory, orders in the manufacturing process, production planning, foreseen demand, etc.

However, unforeseen events in the supply chain could make an initially optimal inventory allocation become less than ideal [1] creating a need for the reallocation of inventory to orders [23]. This situation is even more problematic in the case of multi-line orders, as the inventory must be distributed amongst the different lines of all orders for allocation to be optimal.

Moreover, in some industries, such as the ceramics sector, the need to reallocate inventory is commonplace, even when no unforeseen events have occurred, due to the LHP problem concerning the tones and calibres of a particular product. This makes the process of allocation much more complex and the suitability of this allocation much more variable and subject to the particular circumstances of the company at a given time. This is demonstrated in [30], in which a mathematical model is proposed for the management of LHP in the ceramics sector, in order to reallocate production planned at multiple plants to order lines in an optimal fashion.

There are also other works that study the problem of the LHP, and even in the ceramic sector [31] or [32]. In [33] a review of the works that propose mathematical models to support the order promising process in LHP situations is made. Subsequently, in [34] and [35] Fuzzy tools are introduced to improve the proposed solutions. These works deal with the process of committing orders, considering the quantities included in planned production orders for different future time periods, and taking into account supply networks with several production plants. But this is a production planning for the future, which can subsequently differ from the final result and therefore does not reflect the real final situation. Especially in cases where the LHP occurs and it is impossible to know the result that will actually come out of production, compared to what has been planned to manufacture. On these occasions, it is necessary to reallocate the inventory in stock, physically existing in the warehouse, to the order lines, once the production has already been carried out, in order to optimize the profit and the number of orders served.

This reallocation is something daily, which must be done quickly, when orders are going to be served, and can change from one day to another, for example, with the arrival of new orders or real units produced, an order cancellation, a break in the warehouse, etc. It is a much more operational process than those that have been analysed in the works found, and that is currently done manually by workers who are explicitly dedicated to this reallocation process.

When there are many orders and also multiple lines, this process is very complex and a tool to manage it in the best possible way, with extreme agility, is required. That is the main objective of this work, to provide companies in which LHP exists a tool that can facilitate the reallocation of existing inventory in the warehouse to the line items optimally and also accompany that tool with a methodology so that can be implemented and exploited in a real environment, in an integrated way with its information system, which is expected to be an ERP.

The studies found in the literature review propose mathematical models as DSSs and they may be of great use for management purposes. But for them to be of practical use, it must be possible that the mathematical model be automated and integrated into the company’s management system. And this aspect is not considered in the works found in relation with the LHP.

For this reason, this study presents a computer application and a methodology which enables the collation of the information necessary and the integration of a mathematical model with the IT systems the company employs. The latter are typically enterprise resource planning (ERP) systems, currently the most widely used IT systems by companies [36; 37] and [38]. Thus, a powerful DSS for the reallocation of inventory to orders in LHP situations can be made available, one which is integrated into companies’ typical management systems and is therefore easy to use and implement in a real setting.

Therefore, this work differs from the others published in relation to the LHP in two principal aspects. First, it does not attempt to allocate planned quantities of inventory in production, since that planning will not be fulfilled due to the effect of LHP, but instead focuses on reallocating the existing inventory optimally, once it is manufactured and its specific characteristics are known. Second, this work, unlike the rest, presents a methodology and a tool that can integrate the use of a mathematical model for the reassignment of existing inventory in the warehouse to the customer order lines that will be used, in environments of LHP. The tool allows integration with the Information System that the company can use. Consequently, it does not focus on proposing a theoretical mathematical model, but proposes the entire solution of the problem for its implementation in a real business environment. Its main characteristics are the simplicity and the compatibility with the Enterprise Resource Planning (ERP) framework, thus making it interesting also from an industrial point of view.

## Description of the LHP problem in the ceramic sector

Ceramics companies manufacture their goods mainly on a make-to-stock (MTS) basis, for reasons which include the considerable setup times involved and technological questions which demand that a minimum amount of product must be manufactured in order to ensure a particular level of homogeneity. In general, in companies of this type, customer orders are made up of several order lines in which quantities of different products are requested. When a customer makes an order inquiry to such a company, normally the Sales Department checks the availability of the requested products. If there is sufficient stock available for the date requested by the customer, the corresponding reservation can then be made (ATP allocation of the order), with a commitment thus being established with the customer.

However, this ATP allocation of an order, initially feasible because the on-time delivery of all the agreed-to orders is possible, may later become impracticable for various reasons [2]. This is a particularly critical situation for the ceramics sector due to LHP, which leads to the appearance of discrepancies between the planned quantities and those that are actually produced. The problems that LHP causes in ceramics companies principally concern the tone and calibre of the products manufactured (although it can also affect their surface appearance and flatness, according to [39]). For the customer to receive a homogeneous product and thus be able to use, place or display different product units together without any undesirable aesthetic effects, the products must be classified according to the tones and calibres they possess. Thus, a planned production of a batch of a single type of product may result in several combinations of tones and calibres being produced (sub-batches) which should not be mixed when allocating inventory to a single order line. This restriction on order fulfilment may mean that some inventory reserves which had been allocated to particular order lines may ultimately prove to be insufficient, as the number of homogeneous units produced is fewer than expected.

The reallocation of inventory to orders may help to resolve this situation. Reallocation involves seeking another manner of allocating products to orders in a way that enables one or more of the following objectives to be achieved: maximisation of profit, compliance with agreed delivery dates, more efficient use of current or future inventory, or lower costs.

In ceramics companies, however, the performance of this reallocation is made much more complex due to the LHP issue. The first reason for this is that any reallocation must ensure that products of different tones and calibres (sub-types) are not mixed together in the same order line, and yet, in order to fulfil an order, all of the order lines must be fulfilled. The second reason is that LHP has a fragmenting effect on the inventory. This is because a production batch of a certain size, in which the tone and the calibre should be exactly the same for that particular product, may need to be broken up into two or more sub-batches of a smaller size, according to the different combinations of tone and calibre ultimately present [40,41]. It means that there are more possibilities to consider when evaluating how to fulfil orders. Logically, the complexity of the problem will increase in proportion to the number of orders received at the time of the reallocation, the number of order lines for each order, and the number of products and different sub-types involved in the inventory reallocation. This increasing complexity justifies the use of a DSS, such as a mathematical programming model. However, a mathematical model could solve the problem from a theoretical point of view, but to use it in a real business environment, managing the reallocation needed in a real ceramic company, a mathematical model must be integrated with its information system, which is usually an ERP [42] and [43]. If the inventory reallocation process is not integrated with the ERP, and there is not a fluent communication between the model and the ERP, the information may not be up-to-date, and a situation may arise where orders cannot be served, or deliveries or invoices are made in the wrong way. All this in addition to the duplicity of systems, with the additional work and the amount of errors that may appear. Therefore, this is a fundamental factor to solve the problem.

**Fig 1:**
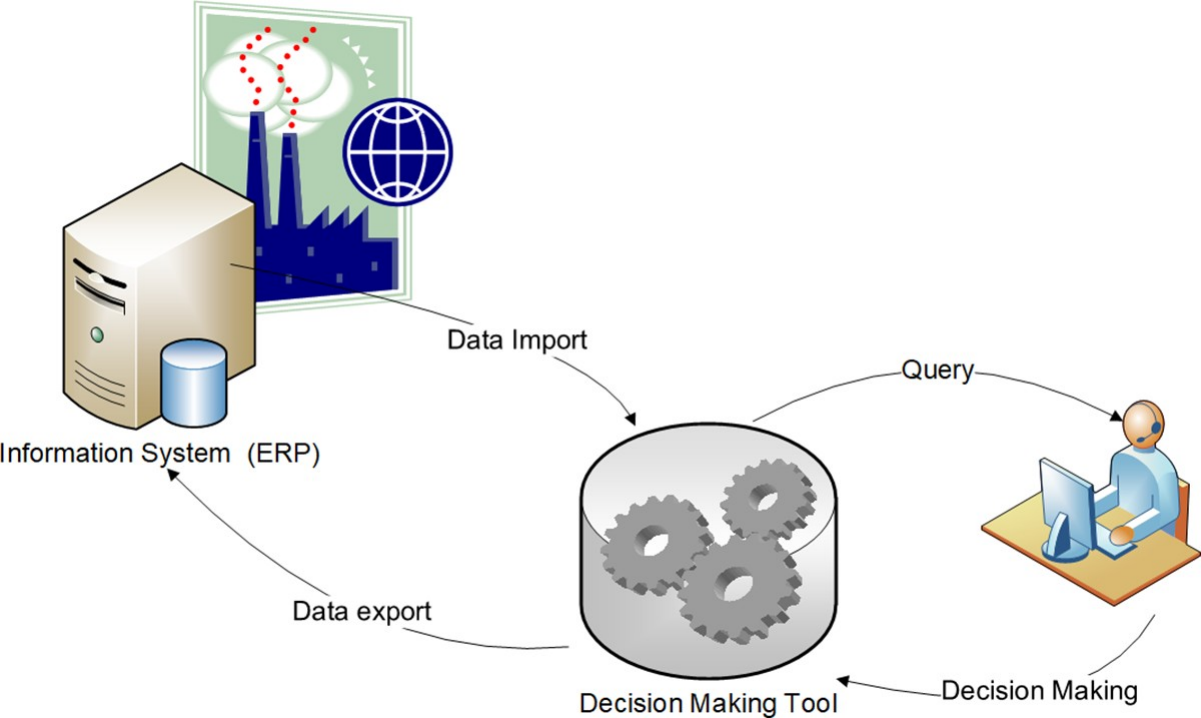
Process for the decision-support system.

The integration of DSS model with the ERP could be carried out as shown in the following image (Fig 1). For this execution, the DSS needs data, which must be in the company's information systems and have to be imported for their use. Subsequently, once executed the decision making tool, where a mathematical model should be implemented, the result of the DSS must be reviewed by a user, who after analysing the existing possibilities, modifies the appropriate parameters in the decision making tool if necessary and makes the final decision. The decision must go from the DSS to the information systems of the company, so that decision can be carried out in the business processes. In order to do that, data must be exported form de DSS to the ERP.

It should be added that this inventory reallocation is a process that must be done on a daily basis, since every time new sales orders are entered and the stock changes, the optimal allocation of inventory to orders changes too, and a new reallocation must be done. Therefore, it should be a very agile and easy to handle process. But nowadays this does not happen. In fact, after analysing how the reallocation is carried out in different companies of the Spanish ceramic sector, it is observed that it is carried out manually, being very difficult to manage, and consuming a lot of time and resources. In addition, this reallocation is mainly based on the experience of the person performing the process. Then, the reallocation may be inappropriate, or based on subjective criteria, and generates a dependency that is not advisable. Furthermore, the method followed by companies to carry out the reallocation varies greatly between them. Thus, there are companies that do the reallocation directly in the ERP order lines, while others do so using an auxiliary excel sheet (whose information is later transferred to the corresponding ERP or not, depending on the case). Other companies make the reallocation directly from the warehouse exit, when loading the trucks and it is not updated in the ERP in any moment.

### Proposed methodology for the reallocation of inventory to orders in LHP situations (MERIO-LHP)

**Fig 2:**
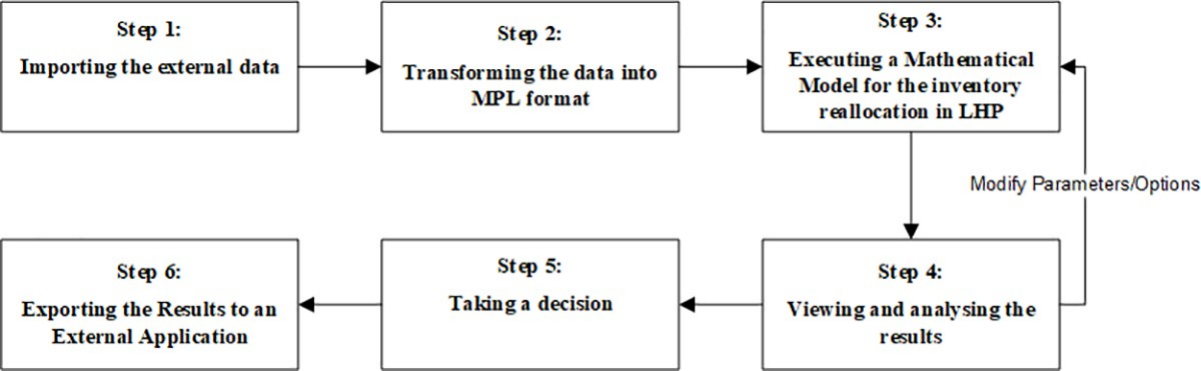
Six step of MERIO-LHP methodology for the reallocation of inventory to orders.

In this point, a Methodology for the reallocation of inventory to orders in LHP situations (MERIO-LHP) is proposed. Following this methodology, any company can make a reallocation of inventory to orders integrated with its information system and based on a mathematical model and objective criteria. This will avoid a lot of unnecessary work, duplicity of data, errors, and the dependence of a specific person. This methodology is composed of six steps as can be shown in the next figure (Fig 2), and is independent of the information system that the company may be using, so it can be used in any business environment. In the next paragraphs, each step of the methodology is briefly explained. Subsequently, the following section presents a case study where a model-based DSS was developed to support this methodology in a real situation.

#### Step 1: Importing the external data

The first step to take is to extract all the necessary data from the information system of the company (whatever it is). These data include information about: Sales Orders, Sales Orders Lines, Inventory, Customers, Items, Prices, etc –…

#### Step 2: Transforming data to the appropriate format

The information extracted from the management systems of the company will be in the format used and defined in it. This format may not be adequate to feed the mathematical model so that it can be executed. Therefore, once you have all the information with which you want to do the reallocation, the second step is to give it an appropriate format so that it fits in the mathematical model.

#### Step 3: Executing a Mathematical Model for the inventory reallocation in LHP

The third step is to execute a mathematical model in order to improve the inventory allocation (making a reallocation) taking into account all the defined parameters, sets, constraints, etc., and to achieve the best solution given the existing data. In this case, in the following section we present a mathematical model for the case study application. The mathematical model could be different if it was necessary in other real cases; but the methodology would be the same.

#### Step 4: Viewing and analysing the results

Once the model has been executed, a result is obtained where, you can see the value of the objective function, as well as, in this case, the number of complete and incomplete sales orders, the total of euros, which customers are left unserviceable, etc. Then, at this step the solution obtained is analysed, before moving to the next step and taking the decision.

Many times the first solution of the model will be perfect and we can move directly to the next step of the MERIO-LHP. In those cases, it could be said that no decision was needed. However, sometimes, the result of the model is not what the user expected, and the user may want to make modifications in terms of parameters, or for example block some customers or specific orders to be served (or not). Then, the information of the model should be changed to re-execute it, going back to step three of the methodology, and generating new solutions that can be analysed and compared between them. In this way, the user can simulate different scenarios, always based on the real data of the company extracted from the information system, executing the model for each one of them. In these cases, it is necessary to make a decision and select the most appropriate alternative. This will be done in the next step.

#### Step 5: Taking a decision

The decision maker must select the most appropriate alternative from the different existing possibilities generated in steps 3 and 4. To do this, he must be able to compare them in a simple way based on the Total of euros of the order book, amount of complete and incomplete orders (in euros), as well as the number of orders for each case.

With this information, the decision of the most appropriate reassignment is made.

#### Step 6: Exporting the results to an external application

The last step of the methodology, once the decision is taken, is to transfer the new solution of allocation of inventory to orders to the information system, that is, to the ERP. In this way, all processes and activities of the company (movements in warehouse, shipments, truck loading, billing, etc.) can continue their usual dynamics, but with the decided reallocation of inventory to orders and updated data based on the chosen solution.

## MERIO-LHP implementation and development of a model-based DSS: Case study

In order to verify the applicability of the MERIO-LHP, the methodology has been tested on a real company which manufactures ceramic flooring and wall facing products.

In this section, first we describe the empirical context of the case study. After that, the application of the methodology to the case study is presented, following the proposed steps and using a DSS model-decision developed specifically for the MERIO-LHP application. In step 3 the mathematical model is executed and explained. Finally, the results of applying the methodology are presented. The principal aim of the test is comparing the inventory allocation policy of the company, which is First Come First Served (FCFS) with the result of the use of MERIO-LHP and the model-based DSS proposed.

### Empirical context

The methodology is applied in a company of the ceramic sector representative of the business sector. The company manufactures different ceramic floor and facing products. Its manufacturing policy is MTS, and its inventory allocation is based on an FCFS policy, in which each time a sales order is entered with its lines, an existing inventory assignment is made in order to serve that order.

The company receives orders every day. The orders are usually multiline, and with delivery dates that can vary from the immediate delivery on the same day, up to several months. At the arrival of the order, an automatic inventory allocation is made in warehouse for each of its lines. If there is no stock that can be reserved, the requirement is included in the planning of the production, and an estimated date is given. However, as previously explained, the production may not be exactly what was expected, due to the LHP, and that makes the entire reservation and inventory allocation not feasible to serve the orders.

Because of that, the order book and inventory reserve for them is very dynamic and variable, and the inventory allocation becomes really complex. At the time of the case study two people were dedicated 100% of their time to reallocate inventory to orders, trying to find a solution to the different situations that arose each day.

The order book studied in the case study can be considered representative of the commercial activity of the company. The inventory allocation was done on a FCFS basis. In total, there were 2.274 orders and 9.347 order lines. In the order lines there were 2.871 different products including 18.138 different sub-batches (tone-calibre combinations) of those products, with a maximum of 92 and minimum of 1 sub-batch per product. The maximum and the minimum number of order lines were 108 and 1 respectively, and the total sales value of these orders was €8.648.346,05. There were 186 orders within the delivery horizon considered for the study (15 day), with 942 order lines. Of those, 72 lines had no inventory allocated (incomplete lines), meaning that there were 49 orders that could not be fulfilled. The main reason that these lines could not be completed was the LHP characteristic of the ceramics sector, causing there to be discrepancies between the planned quantities of homogeneous products and those actually manufactured, with the latter being fewer in number and more fragmented due to tone and calibre differences. Of the total sales value of the order book, € 4.756.786,71 corresponded to orders which could be fulfilled and € 3.891.559,34 which could not, due to lack of product on one or more lines.

**Fig 3:**
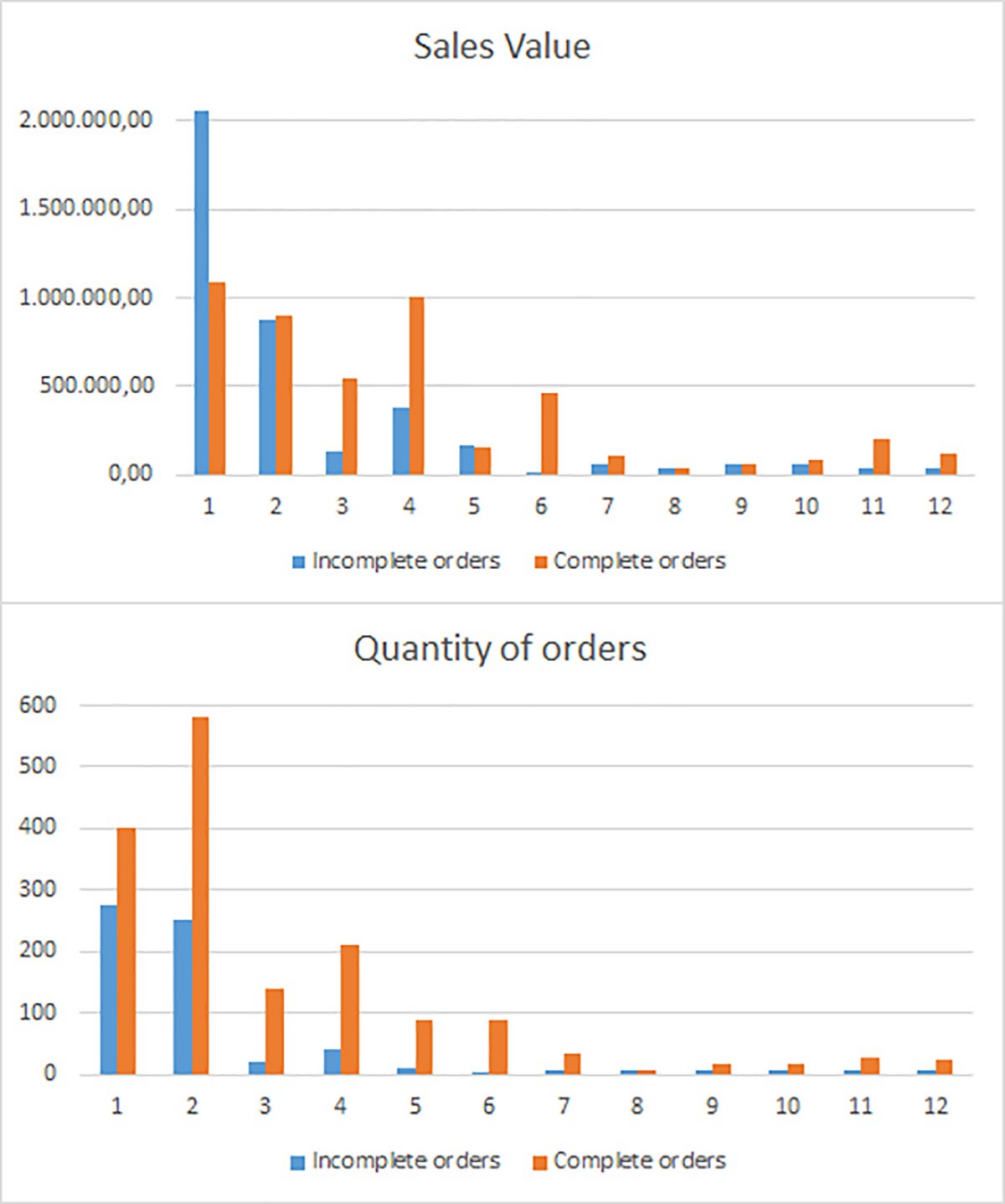
Order situation on an FCFS basis. Sales Value and Quantity of Orders.

Fig 3 shows the situation of the total year order book at the company by months, after allocation of inventory on a FCFS basis. The figure shows two graphs. In the first one, the Sales value (in euros) of the complete and incomplete orders can be seen and compared. In the first month, more than 2.000.000 € correspond to orders that can not be served because they are incomplete, which is about double the amount of the complete ones. The second graph shows the Quantity of orders that are completed or incomplete. In the first month, there are 274 orders incomplete, and nearly 400 completed.

To be able to execute all of the steps of the MERIO-LHP, including the importing and exporting of the data, it was necessary to modify the ERP system used by the company. In this case, the ERP with which integration took place was MS Dynamics Ax. For this purpose, it was necessary to develop the data model in the ERP. This adaptation of the ERP to the specific problems of the ceramics sector represents the first step towards the development of a vertical ERP solution for the ceramics sector. A vertical solution can be defined as “the adjustment, modification or extension of a general ERP solution to the specific business processes of particular industries” [44]. In this way, the implementation of an ERP can be aligned with particular business needs [39]. Currently, there is no vertical ERP solution for the ceramics sector amongst the ERPs marketed by the world’s biggest IT system developers. Therefore, the DSS presented in this article and its adaptation to MS Dynamics AX represent an important step towards the development of such a solution, achieving the fit of ERP with organizational business processes, one of the most important factors for the ERP selection and implementation [45].

### Model-based DSS for MERIO-LHP

Establishing the methodology is necessary to do the reallocation of inventory to orders in LHP situations, but is not enough. To be implemented in the real companies, tools to guide the user to carry out each of the proposed steps are required. In that sense, a model-based DSS was created to help the user to implement the methodology. In the following points, each step of the methodology and the implementation of the model-based DSS in the case study are explained.

#### Step 1: Importing the external data

The first step is to extract the data from the company’s system (the ERP) and introduce them into the model-based DSS tables. The extraction is undertaken using an Open DataBase Connectivity (ODBC) connection, a standard interface with which to access database management systems (DBMSs) and which was developed by SQL Access Group in 1992. This makes it possible to access any data from any application, regardless of the DBMS which stores the data.

Obviously, every time the system is implemented in a different company, with its particular environment, the OBDC connection must be optimised for the application that the company uses. The data that must be imported are those referring to:

- Order headers
- Order lines
- Stock situation
- All of the data imported are stored in tables specifically designed for this purpose in the DSS.

#### Step 2: Transforming data to the appropriate format

Generally, the data imported will not have the required format for the DSS execution. A process through which to transform the data into the correct format is therefore needed. The second step of the process concerns this data formatting, using Visual Basic, by means of which the data imported in Step 1 are transformed (having been stored in the imported data tables), and the data are generated in new tables, which are in the ideal format for use in the mathematical model. The assistant employs a macro written in Visual Basic for this purpose and it carries out various elimination and update queries in the appropriate tables. The result is a set of tables that contain the required data in a format that can be used for the execution of the mathematical model presented in this study.

#### Step 3: Executing a mathematical model for the inventory reallocation in LHP

The mathematical model used for the model-based DSS implemented in the case study is presented in the following paragraphs. This mathematical model has all the components needed to make the reallocation, and is easy to be implemented and used in ceramic SME´s. The objective of the model is to optimize the allocation of inventory to orders, maximizing the total sales value of the orders to be fulfilled and attempting to fulfil the orders with the earliest delivery dates. The mathematical model will take as input the orders that have been submitted to a current inventory whose delivery date is within a certain period of time, called the planning horizon. The larger the planning horizon considered by the decision maker, the greater number of requests is used in the model. Logically, the first days of the planning horizon will contain the orders with the closest or urgent delivery date. These first days, whose exact number determines the decision maker using a parameter, determine the delivery horizon. Within the delivery horizon are the orders that the company must deliver immediately and, therefore, the orders for which it is necessary to try to make the inventory as a priority. For that reason, in the model, two concepts are used: the “planning horizon” and the “delivery horizon”. The “planning horizon” refers to the period of time within which the delivery dates of the orders to be considered in the mathematical model fall. The “delivery horizon” concerns a shorter period of time, in which the delivery of orders is imminent; those orders whose due date for delivery falls within this period must be prepared in the warehouse without delay so that they can be sent out. The model prioritizes the allocation of inventory to orders whose delivery date is within the delivery horizon, given that these orders must be dealt with urgently in the warehouse.

The mathematical model described below is made up of indices, sets, parameters, decision variables, an objective function and constraints.

Table 1 shows indices, sets and parameters, and Table 2 shows decision variables.

**Table 1:** Indices, sets and parameters.

Indices – *i* order – *l* order line – *k* product – *b* sub-batch (each particular combination of a tone and a calibre).	Sets – *I* Set of orders which are within the planning horizon h. – *I(h*_*e*_*)* Set of orders whose delivery dates are within the delivery horizon *h*_*e*_. – *L(i)* Set of order lines *l*, which contain *k* and form part of order *i*. – *B(k)* Set of sub-batches of product k
Parameters – *p*_*1*_ Specific weight given to the sales value of the order in the objective function. – *p*_*2*_ Specific weight given to the delivery date in the objective function. – *b*_*i*_ Total sales value of order *i*. – *h* Planning horizon (expressed in days). – *h*_*e*_ Order delivery horizon (expressed in days). – *fe*_*i*_ Delivery date of order *i*, expressed in days remaining from the moment at which the model is implemented. – *n*_*I(he)*_ Number of orders whose delivery dates are within the delivery horizon (*h*_*e*_). – *nli* Number of order lines in order i. – *d*_*kil*_ Requested quantity of product *k*, in line *l* of order *i*. – *q*_*kb*_ Quantity of product *k* and sub-batch *b* present in the warehouse. – *b*_*max*_ Maximum order value (Maximum of the *b*_*i*_). – *b*_*min*_ Minimum order value (Minimum of the *b*_*i*_). – *ε* Positive value, small and lower than 1. This value is used in order to avoid the possibility that a term in the objective function has a value of zero (for example, 0.001).	

**Table 2:** Decision variables.

Decision variables – *Y*_*i*_ Binary variable indicating whether the order has been completely reserved (i.e. all its lines L(i) are reserved). If this is the case, the value will be taken to be (1) and, if not, the value will be taken as (0). – *U*_*kilb*_ Binary variable indicating whether the line (l) of the order (i) has a product (k) and a sub-batch (b) reserved. If this is the case the value will be taken to be (1) and, if not, the value will be taken as (0). – *ATPO*_*kb*_ Quantity of on-hand stock of the product (k) and sub-batch (b), which is unreserved and available to promise

The basic problem explained may be expressed as the following MILP (Mixed Integer Linear Programming) model:

$$Max\;[z]=p_{1}\sum_{i}\left(\frac{b_{i}-b_{min}+\varepsilon }{b_{max}-b_{min}}\right)Y_{i}+p_{2}\sum_{i}\left(\frac{h-{fe}_{imin}+\varepsilon }{h}\right)Y_{i}$$ (1)

$$\sum_{i\in I(h_{e})}Y_{i}=n_{I(h_{e})}$$ (2)

$$(nl_{i}-\sum_{k}\sum_{l\in (L(i)}\sum_{b\in B(k)}U_{kilb})\leq (1-Y_{i})nl_{i};\forall i$$ (3)

$$\sum_{i}\sum_{l\in (L(i)}d_{kil}U_{kilb}+{ATPO}_{kb}=q_{kb};\forall k,b\in B(k)$$ (4)

$$\sum_{b\in (B(k)}U_{kilb}\leq 1;\forall i,l\in l(i),k$$ (5)

$$U_{kilb},Y_{i}\in \{0,1\};\forall k,i,l,b$$ (6)

$$U_{kilb},Y_{i}\in \{0,1\};\forall k,i,l,b$$ (7)

The first term of the objective function maximises the sales value, while the second minimises the sum of the days remaining for the delivery of all of the orders. There are two weighting factors (p1 and p2), which enable greater weight to be given to one objective or the other. The magnitudes of the two terms of the objective function are very different. The sales value (b_i_) of an order can be several hundred thousand euros, while h and *fe*_*i*_ are expressed in days and their values are obviously much lower. h can be a maximum of 365, assuming that the planning horizon to be considered is one year. *fe*_*i*_ is always lower than h. So the equations presented in this study have been employed in order to bring the two factors within a comparable range.

Constraint (2) ensures that all of the orders in the delivery horizon will be completed, i.e. material will be reserved in order to fulfil all of its order lines. This may not be feasible. In that case, this constraint must be eliminated in order to obtain an optimal result, but not all of the orders will be completed. Constraints (3) ensures that only the completed orders, i.e. those which have material reserved for each and every one of its order lines, will be taken into account in the objective function. Constraints (4) ensures the continuity of inventory, so that the sum of the quantities allocated to the different order lines of product k and sub-batch b, together with the unreserved on-hand stock of that product and sub-batch, is never higher than the quantity of product k and sub-batch b actually present in the warehouse. Constraints (5) limits to 1 the number of sub-batches that can be reserved for an order line. Constraints (6) determines the decision variables as binary variables Ukilb and Yi. Finally, constraints (7) indicates that the on-hand stock of product k and sub-batch b can never be negative.

In the development of the DSS for the application of the methodology in the case study, the mathematical model is executed using the MPL Modeling System program, version 4.2K, 64 bits. The solver used to implement the model in was the Gurobi program, version 4.0.1, also 64 bits.

#### Step 4: Viewing and analyzing the results

In the fourth step, the results obtained from the model can be viewed, along with the orders and order lines that will be fulfilled and the resulting stock situation.

**Fig 4:**
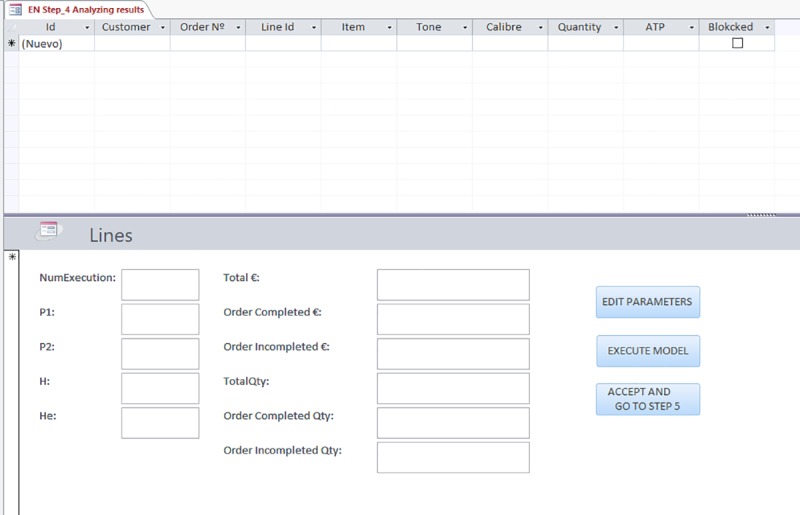
Solution analysis and model changes introduction screen of the DSS.

Different modifications and simulations can be carried out directly in a specific screen of the model-based DSS, which does not interfere with the ERP. The mathematical model can be re-executed with variations, for example by changing the parameters p1 or p2, the horizons taken into account H and He, or even blocking customer orders to be served on a mandatory basis. To carry out this step, a set of queries and processes have been developed allowing the modification of parameters and data to obtain new solutions. Fig 4 shows one of the query screens of the model-based DSS, from which the data of the lines and their assignment can be consulted, and parameters and data of the model can be modified to create new scenarios and solutions. The model can be executed again with the changes made pressing a button.

#### Step 5: Taking a decision

**Fig 5:**
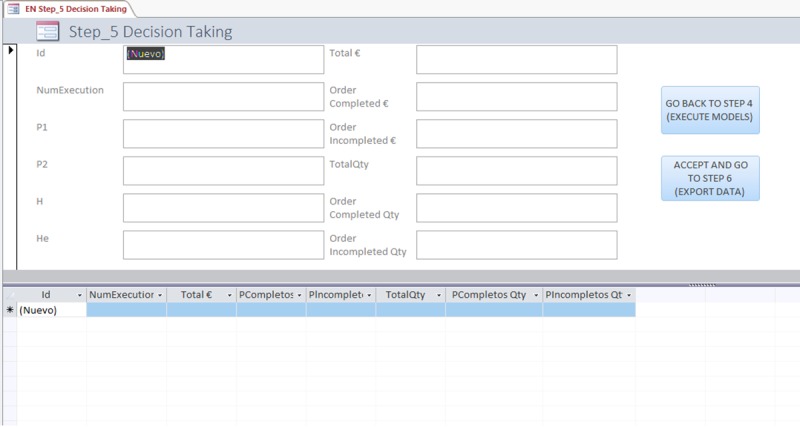
Compare solutions screen of de model-based DSS.

After viewing the results, the fifth step concerns taking the appropriate decisions. The following image, Fig 5, shows the “Control panel” used for decision making. In the lines you can consult the results of the different executions of the model, and when the line is selected, the parameters used for that specific execution.

Once all the results obtained have been reviewed, and the decision has been taken to improve the reassignment of inventory to orders, the line is selected and the "go to step 6" button is pressed. Alternatively, it is possible to go back to step 5 and re-modify model parameters to obtain new results.

#### Step 6: Exporting the results to an external application

Once the decisions have been taken, the only remaining task is to export the data to the company’s management system. To do this, an ODBC connection is established which transfers the data to the tables of the ERP system or another system which the company uses. A prior conversion of the data into the appropriate format must take place. This can be undertaken using a macro written in Visual Basic.

### Results of the case study

**Fig 6:**
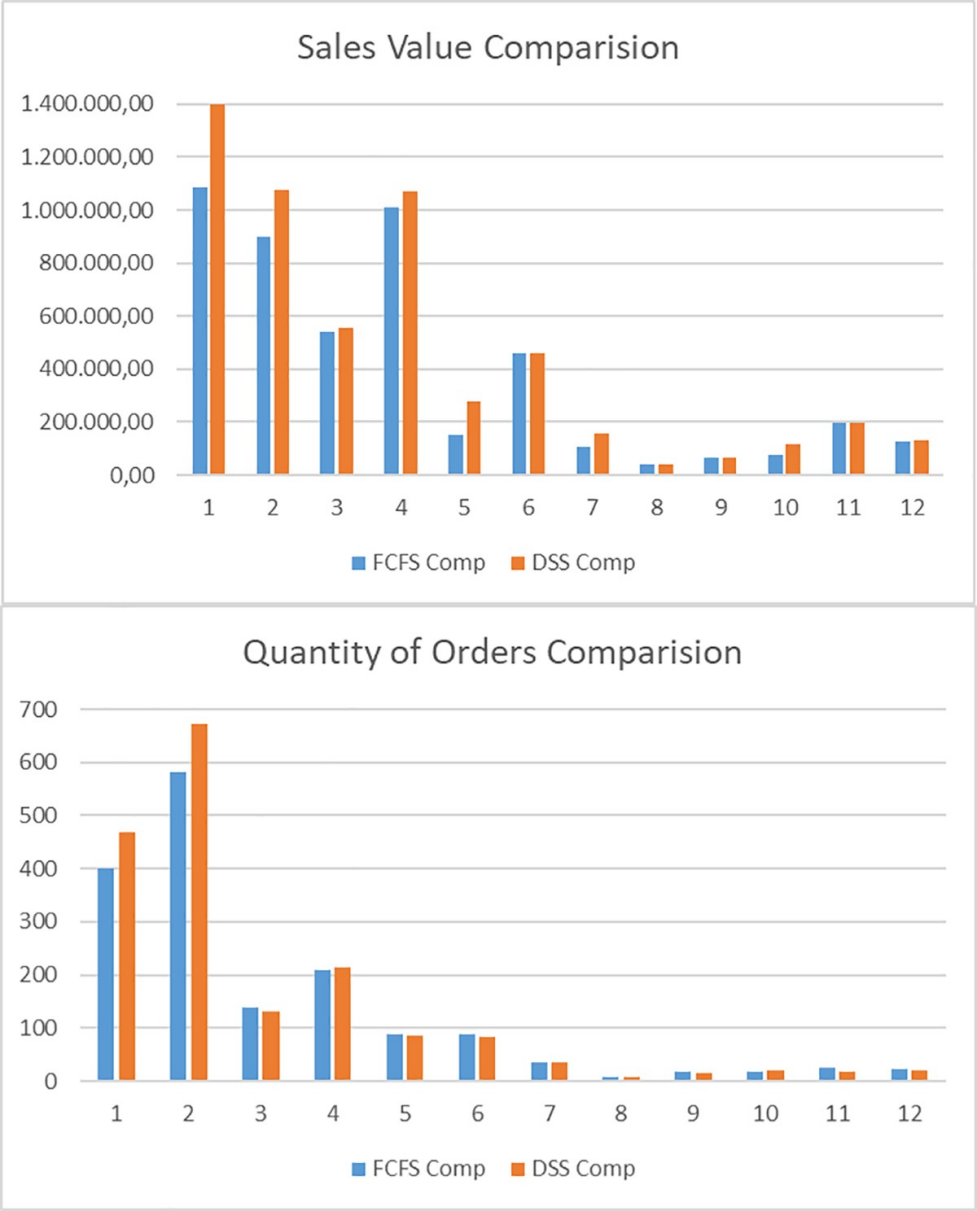
Comparison of the completed orders using an FCFS strategy with DSS optimization. Sales Value and Quantity of Orders.

**Fig 7:**
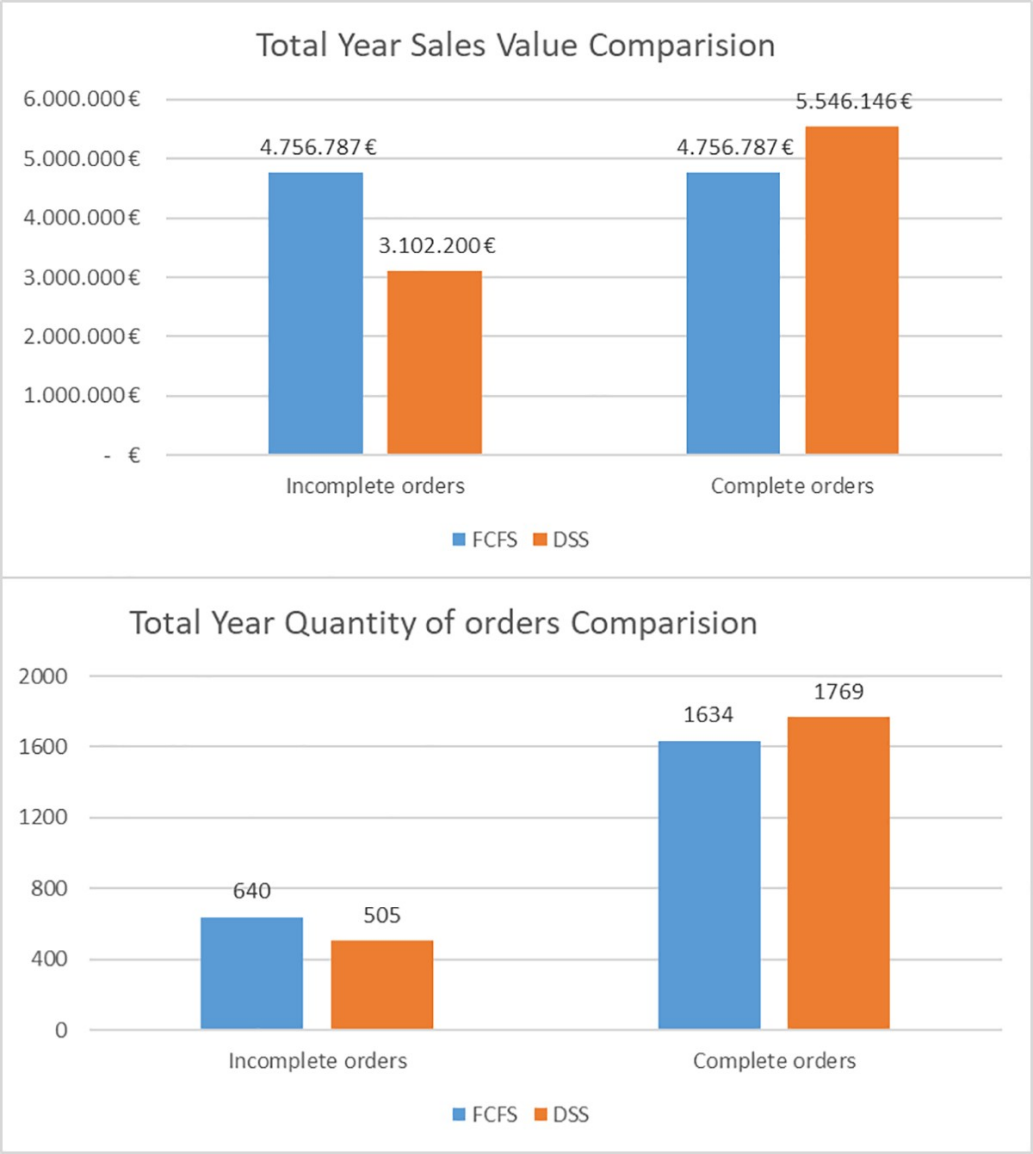
Comparison of the complete and incomplete orders using both strategies (FCFS and DSS) per year. Sales Value in Euros.

The order book of the case study was then put through the DSS for the inventory reallocation. In order to analyse the improvement obtained with the DSS, a comparison of the initial situation, following a FCFS policy, and the result after applying the MERIO-LHPO and the DSS was made. The results per month can be seen below in Fig 6 and Fig 7 shows the total improvement result, for the whole year.

As can be seen, there is an overall improvement in the allocation of inventory to orders across the order book, as 135 more orders can be fulfilled (a 6%) with an increase of 789.359,57€ in sales value (a 9%).

In the short term, the improvements obtained by the model are even greater. For the first two months (January and February), there are increases of 10% and 11% in the number of orders which can be fulfilled, which translates into an increase in turnover of 10% in both cases. The reason for this is that inventory allocations for orders that will be fulfilled in the long term are replaced with allocations for orders that must be fulfilled in the short term. Therefore, with the DSS optimal allocations of inventory to orders based on the objectives set in the mathematical model become possible, also enabling the decision maker to simulate different scenarios in a quick and straightforward manner.

## Conclusions

LHP is characteristic of the ceramics industry, where the manufacturing process generates products with lack of homogeneity with regard to tone and calibre. However, a single customer order must be fulfilled with homogeneous products. Therefore, the correct allocation of the different tone-calibre combinations of a product held in the inventory to the different sales orders received is essential, greatly increasing the complexity of the allocation of inventory to orders. Moreover, the presence of LHP may mean that what at one time may have been an optimal inventory allocation can become less than ideal, making an inventory reallocation necessary. This creates a problem, which is difficult to manage, making a DSS to be required.

In this study, the problems caused by LHP for the allocation of inventory to orders in the ceramics sector have been described. To solve them, a mathematical model can be used, but in the real world, it is not sufficient, and users need tools and procedures to carry out their work in an efficient manner. In that sense, the authors of this work propose the methodology MERIO-LHP to manage the reallocation to orders in LHP situations. In addition, to make possible the implementation of this methodology in a real business environment, a model-based DSS has been developed and presented. This model-based DSS can be executed easily, following the six steps proposed in MERIO-LHP and can be integrated with the management information systems that companies’ use, such as ERP systems. In this way, a powerful DSS system capable to reallocate inventory to orders in LHP situations has been obtained. Furthermore, the MERIO-LHP and the model-based DSS have been validated when applied to a real setting in the ceramics manufacturing industry: it has been shown that the DSS functions correctly and is capable of finding optimal solutions which make it possible for both incomes per sale and the ratio of order fulfilment to be improved, in comparison with the usual method of allocation, which is performed on a FCFS basis.

Future lines of research in relation with this work could be the inclusion in the model-based DSS of customer’s classification, priority of orders, partial deliveries, or study the effects of size of order lines and sub-batches on reallocation efficiency, and on the number and size of the remaining sub-batches of items in the warehouse after the reallocation. The study of LHP could also be extended to other business areas, such as forecast calculations, stock management or production management.

## Supporting information

S1 File SalesOrder. (XLSX)

S2 File SalesLines. (XLSX)

S3 File Inventory. (XLSX)

S4 File Products. (XLSX)

## References

[1] Vollmann TE, Berry WL, Whybark DC. Sistemas de Planificación y control de la fabricación. 1st ed. Irwin / McGraw-Hill 1995.

[2] Alarcón F, Alemany MME, Lario FC, Oltra-Badenes R. La falta de homogenidad del producto (FHP) en las empresas cerámicas y su impacto en la reasignación del inventario, Boletín de La Sociedad Española de Cerámica y Vidrio. 2011; 50 (1), 49–58.

[3] Wanke P, Henrique Alvarenga H, Correa H, Hadi-Vencheh A, Kalam Azad A. Fuzzy inference systems and inventory allocation decisions: Exploring the impact of priority rules on total costs and service levels. Expert Systems with Applications. 2017; 85 (1), 182–193.

[4] Jonsson H, Silver EA. Stock allocation among a central warehouse and identical regional warehouses in a particular push inventory control system. International Journal of Production Research. 1987: 25 (2), 191–205.

[5] Eren B, Chan Y. A Combined inventory and lateral re-supply model for repairable items—Part I: Modeling an Air Force logistics problem In Zeimpekis V., Kaimakamis G., & Daras N. J. (Eds.), Military logistics: Research advances and future trends. Springer International Publishing 2015.

[6] Perez-Rodriguez N, Holguin-Veras J. Inventory-allocation distribution models for postdisaster humanitarian logistics with explicit consideration of deprivation costs. Transportation Science. 2015; 30 (4), 680–708.

[7] Wu HH, Yeh CS. A study of the bin inventory allocation model for LED-CM plants. Applied Mechanics and Materials. 2014; 543–547, 4440–4443.

[8] Wu HH, Jiang XY. Improved genetic algorithms for optimization of inventory allocation in LED chip manufacturing plants, Journal of Interdisciplinary Mathematics. 2017; 20:3, 727–738

[9] Kristianto Y, Gunasekaran A, Helo P, Hao Y. A model of resilient supply chain network design: A two-stage programming with fuzzy shortest path. Expert Systems with Applications. 2014; 41 (1), 39–49.

[10] Protopappa-Sieke M, Sieke MA, Thonemann U. Optimal two-period inventory allocation under multiple service level contracts. European Journal of Operational Research. 2016; 252 (1), 145–155.

[11] Luo K, Bollapragada R, Kerbache L. Inventory allocation models for a two-stage, two-product, capacitated supplier and retailer problem with random demand. International Journal of Production Economics. 2017; 187, 168–181.

[12] Zhao H, Huang E, Dou R, Wu K. A multi-objective production planning problem with the consideration of time and cost in clinical trials. Expert Systems with Applications. 2019; 124, 25–38.

[13] Kang K, Pu W, Ma Y, Wang X. Bi-objective inventory allocation planning problem with supplier selection and carbon trading under uncertainty. PLoS ONE. 2018; 13 (11) 10.1371/journal.pone.0206282 PMC6261456 30485273

[14] Esmaeili-Najafabadi E, Fallah Nezhad MS, Pourmohammadi H, Honarvar M, Ali Vahdatzad M. A joint supplier selection and order allocation model with disruption risks in centralized supply chain, Computers & Industrial Engineering. 2019; 127,734–748.

[15] Chen CM. Inventory Allocation in the Presence of Service-Level Agreements. Production and operations management, 2018; 27 (3), 553–577.

[16] Chen CZ, Zhao ZY, Ball MO. Quantity and Due Date Quoting Available to Promise. Information Systems Frontiers. 2001; 3(4) 477–488.

[17] Chen CY, Zhao Z, Ball MO (2002) A Model for Batch Advanced Available-To-Promise, Production and Operations Management. 2002; 11 (4), 424–440.

[18] Pibernik R. Advanced available-to-promise: Classification, selected methods and requirements for operations and inventory management. International Journal of Production Economics. 2005; 93–94, 239–252.

[19] Pibernik R. Managing stock-outs effectively with order fulfilment systems», Journal of Manufacturing Technology Management. 2006; 17 (6) 721–736.

[20] Ball MO, Chen CY, Zhao ZY. (2004). Available to promise Handbook of quantitative Supply Chain Analysis: Modeling the E-business Era. 1st ed. Boston: Kluwer Academic Publishers.

[21] Meyr H. Customer segmentation, allocation planning and order promising in make-to-stock production. OR Spectrum. 2009; 31 (1) 229–256.

[22] Pibernik R, Yadav P. Inventory reservation and real-time order promising in a Make-to-Stock system, OR Spectrum. 2009; 31 (1) 281–307.

[23] Xu PJ, Allgor R, Graves SC. Benefits of Reevaluating Real-Time Order Fulfillment Decisions, MSOM—Manufacturing & Service Operations Management. 2009; 11 (2).

[24] Venkatadri U, Srinivasan A, Montreuil B, Saraswat A. Optimization-based decision support for order promising in supply chain networks, International Journal of Production Economics. 2006: 103 (1) 117–130.

[25] Xiong MH, Tor SB, Bhatnagar R, Khoo LP, Venkat S. A DSS approach to managing customer enquiries for SMEs at the customer enquiry stage, International Journal of Production Economics. 2006; 103 (1) 332–346.

[26] Pajouh FM, Xing D, Zhou Y, Hariharan S, Balasundaram B, Liu T, Sharda R. A Specialty Steel Bar Company Uses Analytics to Determine Available-to-Promise Dates INFORMS Journal on Applied Analytics. 2003; 43 (6) 503–517

[27] Yang W, Fung RYK. An available-to-promise decision support system for a multi-site make-to-order production system. International Journal of Production Research. 2014; 52 (14) 4253–4266

[28] Castiglione C, Alfieri A, Pastore E. Decision Support System to balance inventory in customer-driven demand, IFAC-PapersOnLine. 2018; 51 (11) 1499–1504.

[29] Mhiri E, Jacomino M, Mangione F, Vialletelle P, Lepelletier G. Finite capacity planning algorithm for semiconductor industry considering lots priority. IFAC-PapersOnLine. 2015; 48(3), 1598–1603.

[30] Alemany MME, Lario FC, Ortiz A, Gómez F. Available-To-Promise modeling for multi-plant manufacturing characterized by lack of homogeneity in the product: An illustration of a ceramic case, Applied Mathematical Modelling. 2013; 37 (5) 3380–3398.

[31] Alemany MME, Ortiz A, Boza A, Fuertes-Miquel VS. A model-driven decision support system for reallocation of supply to orders under uncertainty in ceramic companies, Technological and Economic Development of Economy. 2015; 21:4, 596–625. 17.

[32] Alemany MME; Grillo H; Ortiz A; Fuertes-Miquel VS. A fuzzy model for shortage planning under uncertainty due to lack of homogeneity in planned production lots. Applied Mathematical Modelling 2015; 39 (15) 4463–4481

[33] GrilloH Alemany MME, Ortiz A. A review of mathematical models for supporting the order promising process under Lack of Homogeneity in Product and other sources of uncertainty. Computers & Industrial Engineering. 2016 Volume 91, 2016, pp. 239–261.

[34] Mundi MI, Alemany MME, Poler R, Fuertes-Miquel VS. Fuzzy sets to model master production effectively in Make to Stock companies with Lack of Homogeneity in the Product. Fuzzy Sets and Systems. 2016; 293 95–112.

[35] Grillo H, Alemany MME, Ortiz A, Mula J. A Fuzzy Order Promising Model With Non-Uniform Finished Goods. International Journal Of Fuzzy Systems. 2018; 20 (1) 187–208

[36] Botta-Genoulaz V, Millet PA. A classification for better use of ERP systems. Computers in Industry. 2005; 56, (6). 573–587.

[37] Grabot B, Botta-Genoulaz V. Special issue on Enterprise Resource Planning (ERP) systems, Computers in Industry. 2005; 56 (6) 507–509.

[38] Oltra-Badenes R, Gil-Gomez H, Bellver R. Factores diferenciales entre los ERP de software libre (FSw ERP) y los ERP propietarios. Dirección y Organización. 2011; (44) 64–73

[39] Millet PA. Toward a model-driven, alignment-oriented ERP methodology, Computers in Industry. 2013; 64 (4) 402–411.

[40] Segura B, Vallada E, Maroto C, Ruiz B. Análisis del sistema de operaciones en empresas del sector cerámico español, Boletín de La Sociedad Española de Cerámica y Vidrio. 2004; 43 (6) 929–932.

[41] Tortajada I, Peris-Fajarnes G, Aguilar M, Latorre P. Análisis del proceso de clasificación cerámica, Boletín de La Sociedad Española de Cerámica y Vidrio. 2006; 45 (1) 22–27.

[42] Botta-Genoulaz V, Millet PA, Grabot B. A survey on the recent research literature on ERP systems. Computers in Industry. 2005; 56 (6) 510–522.

[43] Gil-Gomez H, Arango MD, Oltra-Badenes R. Evolutions and Trends of Information Systems for Business Management: the M-Business. A Review. Dyna, 2010; 77 (163) 110–125.

[44] Oltra-Badenes R, Gil-Gomez H, Bellver-Lopez R, Asensio-Cuesta S. Análisis de requerimientos funcionales para el desarrollo de un ERP adaptado a la gestión de la logística inversa, Dirección y Organización. 2013; 49, 5–16.

[45] Zeng YR, Wang L, Xu XH. An integrated model to select an ERP system for Chinese small- and medium-sized enterprise under uncertainty. Technological and Economic Development of Economy. 2013; 23(1), 38–58.

